# DNA methylation of the INSR gene as a mediator of the association between prenatal exposure to famine and adulthood waist circumference

**DOI:** 10.1038/s41598-020-69120-w

**Published:** 2020-07-22

**Authors:** Zhenghe Wang, Jieyun Song, Changwei Li, Yanhui Li, Luqi Shen, Bin Dong, Zhiyong Zou, Jun Ma

**Affiliations:** 1 Department of Epidemiology, School of Public Health, Southern Medical University, Guangzhou, China; 2 Institute of Child and Adolescent Health, School of Public Health, Peking University, Beijing, 100191 China; 3 Department of Epidemiology and Biostatistics, University of Georgia College of Public Health, Athens, GA USA; 4 National Health Commission Key Laboratory of Reproductive Health, Peking University Health Science Center, Beijing, 100191 China

**Keywords:** Methylation analysis, Obesity, Epidemiology

## Abstract

The aims of this study were to explore whether DNA methylation at *INSR* and *IGF2* mediated the association of prenatal exposure to the Chinese great famine with adulthood waist circumference (WC) and BMI. A total of 235 subjects were selected into the present study from severely affected province and a neighbor province with less severely affected famine in China through multi-stage clustered random sampling. DNA methylation at the *INSR* and *IGF2* gene promoter regions was detected by the Sequenom’s MassARRAY system. The “mediation” package of R was used to evaluate the mediation effect of DNA methylation on the association between prenatal exposure to the famine and adult WC and BMI. The results showed that prenatal famine exposure was significantly associated with higher overall methylation level of the *INSR* gene (*d* = 3.6%; 95% CI 1.2–6.0; *P* = 0.027) and larger adulthood WC (*d* = 2.72 cm; 95% CI 0.20–5.24; *P* = 0.034). Furthermore, famine significantly increased methylation levels at four CpG sites. Methylation of the CpG7 site mediated 32.0% (95% CI 5.0–100.0%, *P* = 0.029) of the association between prenatal exposure to the Chinese great famine and adulthood WC. In conclusion, Epigenetic changes to the *INSR* might mediate the adverse effect of prenatal famine exposure on WC in adulthood.

## Introduction

Obesity is a major risk factor for cardiometabolic disorders including cardiovascular disease, stroke, and diabetes^1,2^. According to the World Health Organization, over 600 million adults are obese worldwide^3^. In China, more than 38% of the total population were overweight or obese in 2011, which translates to 24.35 billion Yuan annual cost^4^.

Over the past four decades, epidemiological studies have observed that prenatal exposure to adverse environment was associated with higher risk of diseases in later life^5^. Epigenetic changes such as DNA methylation induced by the prenatal adverse environmental exposure might be a potential mechanism underlying the association^6^. DNA methylation features affect the transcription of gene(s) in nearby regions. Once changed, the methylation features can persist and lead to long term consequences^7^. Animal studies found that maternal exposure to malnutrition during gestation modified DNA methylation of certain genomic regions in offspring, and the methylation was subsequently associated with poor phenotypes in later life. Furthermore, the changes in DNA methylation level even continued to the third generation^8–10^. In human, prenatal exposure to the six months’ Dutch famine was associated with changes of DNA methylation features in genes, including *INSR*, *CPT1A*, and *IGF2,* which are involved in growth and metabolism^11,12^. A recent genome-wide epigenetic study of the Dutch famine reported that DNA methylation mediated associations between prenatal exposure to the famine and body mass index (BMI) in adulthood^13^. However, these findings are only from European, lacking replication in other races.

As the largest famine that has been well documented in human history, the 1959–1961 Chinese great famine was featured by its long duration (3 years) and extreme severity. As reported in our previous publication, 11.6% of the middle-aged and older Chinese adult had immediate family member(s) starved to death during 1959–1961^14^. The Chinese great famine provided an opportunity to explore the mediation effect of DNA methylation in the association between prenatal famine exposure and adverse phenotypes in later life. Previously, we have reported that early-life severe famine exposure might increase methylation level in the *IGF2* and *INSR* genes, and the change in the *IGF2* gene was also linked with higher level of total cholesterol in adulthood^15,16^.

Waist circumference (WC) reflects central obesity, and has been suggested to be superior to body mass index (BMI), particularly among Asian populations, in predicting cardiovascular disease risk^17^. Thus, WC may be a better indicator to reflect the effect of early-life famine exposure on adult risks of non-communicable chronic disease. In this context, we explore the impact of the Chinese great famine on central obesity and the potential mediation effect of gene methylation level on the famine-obesity association among participants of the Genomic Research of the Chinese Famine (GRECF) study.

## Results

Characteristics of study participants are presented in Table 1. A total of 75 participants had prenatal famine exposure, and 160 were not exposed to famine during gestation. Mean age of the non-exposed group was similar to the prenatal famine exposed group (*P* = 0.149). Compared with the non-exposed group, participants in the famine exposed group had a higher waist circumference (85.86 vs. 82.97 cm, *P* = 0.029). However, we did not observe significant differences in BMI, physical activity, smoking, drinking, or frequencies of meat, vegetable, fruit, and milk consumption between the two groups.

**Table 1:** Characteristics of study participants.

Variables	Non-exposed group (n = 160)	Prenatal famine exposed group (n = 75)	*P*
Age (mean ± SD), years	54.6 ± 2.6	55.1 ± 0.8	0.149
WC (mean ± SD), cm	82.97 (9.78)	85.86 (10.57)	0.029
BMI (mean ± SD), kg/m^2^	24.27 (3.85)	24.43 (3.2)	0.737
Male, n (%)	80 (50.0)	37 (50.7)	0.924
PA level, n (%)			0.621
Light PA	119 (74.4)	60 (80.0)	
Moderate/vigorous PA	41 (25.6)	15 (20.0)	
**Frequencies of food consumption**			
Meat, n (%)			0.914
< 1 times/day	106 (66.3)	47 (62.7)	
≥ 1 times/day	54 (33.8)	28 (37.3)	
Vegetable, n (%)			0.365
< 1 times/day	8 (5.0)	6 (8.0)	
≥ 1 times/day	152 (95.0)	69 (92.0)	
Fruit, n (%)			0.645
< 1 times/day	101 (63.1)	45 (60.0)	
≥ 1 times/day	59 (36.9)	30 (40.0)	
Milk, n (%)			0.472
< 1 times/day	138 (86.3)	62 (82.7)	
≥ 1 times/day	22 (13.7)	13 (17.3)	
**Lifestyle behaviors**			
Smoking, n (%)			0.665
< 400 cigarettes	107 (66.9)	48 (64.0)	
≥ 400 cigarettes	53 (33.1)	27 (36.0)	
Drinking, n (%)			0.071
Current drinking	105 (65.6)	40 (53.3)	
Non-current drinking	55 (34.4)	35 (46.7)	

*SD* standard deviation, *PA* physical activity, *WC* waist circumference, *BMI* body mass index.

**Figure 1:**
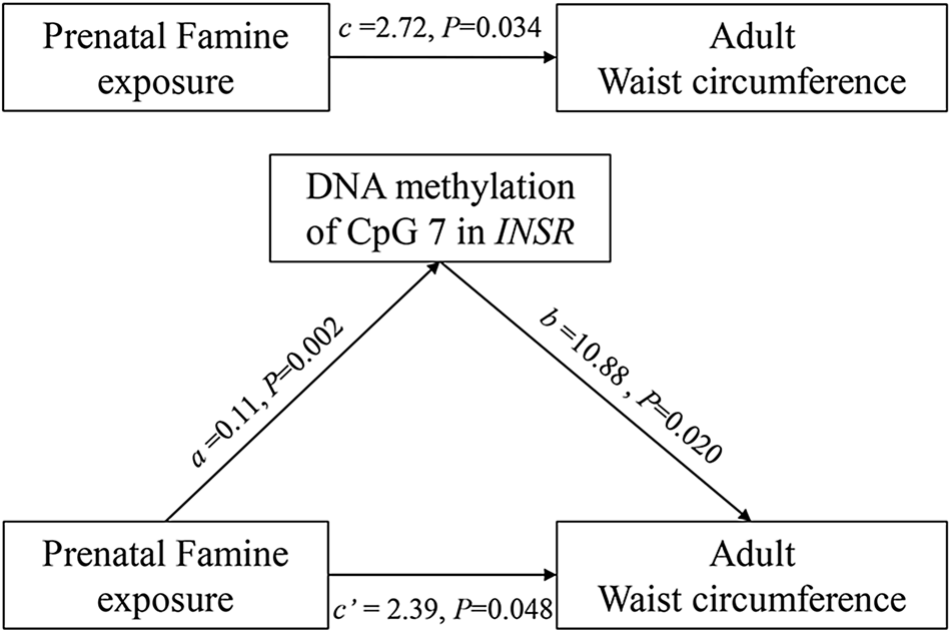
The mediation analysis of DNA methylation at CpG 7 in association between prenatal famine exposure and adult waist circumference. Path (a) was defined as the effect of the independent variable (prenatal famine exposure) on the mediator (DNA methylation of CpG 7 at *INSR*). Path (b) was the effect of the mediator on the dependent variable by controlling the effect of the prenatal famine exposure. Path (c) was the total effect of the independent variable on the dependent variable. Path (c′) shows the direct effect from independent variable to dependent variable. All the analysis adjusted for bisulfite batch, province, gender, physical activity level, the frequencies of meat, vegetables, fruit, milk, smoking and alcohol use.

Multiple adjusted associations between prenatal famine exposure, DNA methylation, and waist circumference and BMI in adulthood were presented in Tables 2 and 3. Participants with prenatal famine exposure had 2.72 cm [95% confidence interval (CI) 0.20–5.24, *P* = 0.034] longer waist circumference compared to those without famine exposure after adjustment of gender, province, smoking, drinking, physical activity, and frequencies of meat, vegetable, fruit, and milk consumption. The effect of prenatal famine exposure on adulthood waist circumference is similar between men and women (Table S1). Meanwhile, prenatal famine exposure was positively associated with the overall DNA methylation level and that at four CpG sites (CpG1, CpG4, CpG5, and CpG7) in the *INSR* gene. Prenatal famine exposure had the greatest impact at the CpG7 site, with the exposed group having 11.2% (95% CI 5.5–16.9%, *P* = 1.4 × 10^−3^) higher methylation level compared to those without famine exposure. DNA methylation at the CpG7 site was also positively associated with waist circumference (Table 3: *β* = 10.88, 95% CI 1.82–19.94, *P* = 0.020). Mediation analysis demonstrated that the mediation path through the CpG7 explained 32.0% (95% CI 5.0–100.0%, *P* = 0.029) of the association between prenatal famine exposure and waist circumference in adulthood (Fig. 1). None of the other CpG sites both in *INSR* and *IGF2* was associated with waist circumference and BMI (Table 3). In addition, the overall DNA methylation differences between prenatal famine exposure group and non-exposed group were larger in the more severely affected province than that in the less affected one (Table S2).

**Table 2:** The association of prenatal exposure to the China famine with WC, BMI and DNA methylation (%).

CpG loci	Difference* (95% CI)	Cohen’s d	*P*	Bonferroni-corrected *P*
WC	2.72 (0.20, 5.24)	0.284	0.034	–
BMI	0.06 (− 0.96, 1.08)	0.016	0.913	–
*INSR*	3.6 (1.2, 6.0)	0.404	0.003	0.027
CpG 1	6.7 (3.1, 10.3)	0.491	3.1 × 10^−4^	2.8 × 10^−3^
CpG 2	3.3 (0.0, 6.6)	0.253	0.052	NS
CpG 3	2.9 (− 0.6, 6.4)	0.213	0.105	NS
CpG 4	6.7 (3.1, 10.3)	0.491	3.1 × 10^−4^	2.8 × 10^−3^
CpG 5	7.6 (3.4, 11.8)	0.486	4.6 × 10^−4^	4.1 × 10^−3^
CpG 6	0.2 (− 4.6, 4.9)	0.018	0.938	NS
CpG 7	11.2 (5.5, 16.9)	0.539	1.5 × 10^−4^	1.4 × 10^−3^
CpG 8	0.3 (− 1.7, 2.2)	0.025	0.799	NS
CpG 9	3.4 (− 0.6, 7.5)	0.377	0.097	NS
*IGF2*	3.2 (0.8, 5.6)	0.443	0.010	0.070
CpG 1	2.4 (− 1.9, 6.7)	0.191	0.276	NS
CpG 2	7.8 (2.9, 12.7)	0.524	0.002	0.014
CpG 3	3.6 (− 0.5, 7.7)	0.330	0.085	NS
CpG 4	1.6 (− 2.3, 5.5)	0.105	0.423	NS
CpG 5	0.2 (− 1.5, 1.1)	0.041	0.754	NS
CpG 6&7	2.5 (0.0, 5.0)	0.055	0.053	NS
CpG 8	0.3 (− 4.1, 4.6)	0.005	0.902	NS

*WC* waist circumference, *BMI* body mass index, *INSR* insulin receptor gene, *IGF2* Insulin growth factor 2 gene, *CI* confidence interval, *NS* not significance, ‘–’ no data.

*Differences between individuals with and without prenatal famine exposure, adjusting for bisulfite batch, province, gender, physical activity level, the frequencies of meat, vegetables, fruit, milk, smoking and alcohol use.

**Table 3:** The association between DNA methylation (%) in the *INSR* and *IGF2* and WC and BMI after adjusted for prenatal famine exposure, respectively.

Gene loci	WC		BMI	
	Βeta (SE)	*P*	Βeta (SE)	*P*
*INSR*	17.29 (11.37)	0.139	0.04 (2.80)	0.989
CpG 1	7.78 (7.31)	0.289	− 0.28 (1.84)	0.878
CpG 2	2.28 (7.74)	0.769	− 1.76 (2.05)	0.391
CpG 3	9.58 (7.93)	0.229	− 1.19 (1.94)	0.541
CpG 4	7.78 (7.31)	0.289	− 0.28 (1.84)	0.878
CpG 5	10.37 (6.25)	0.099	0.77 (1.60)	0.631
CpG 6	− 0.39 (5.95)	0.948	− 0.64 (1.45)	0.657
CpG 7	10.88 (4.62)	0.020	1.56 (1.16)	0.179
CpG 8	− 11.45 (31.58)	0.718	− 12.04 (8.60)	0.164
CpG 9	12.98 (12.99)	0.332	9.63 (3.99)	0.017
*IGF2*	− 10.04 (10.61)	0.346	− 1.01 (4.15)	0.808
CpG 1	− 12.85 (6.73)	0.062	− 0.21 (2.37)	0.930
CpG 2	− 0.67 (5.54)	0.905	0.16 (2.17)	0.942
CpG 3	1.44 (6.32)	0.819	1.70 (2.44)	0.488
CpG 4	− 0.47 (6.71)	0.944	− 3.87 (2.62)	0.143
CpG 5	− 81.03 (53.39)	0.134	− 12.22 (26.07)	0.641
CpG 6&7	− 14.64 (12.34)	0.238	− 8.96 (5.01)	0.076
CpG 8	− 6.40 (5.99)	0.287	− 1.59 (2.35)	0.501

*INSR* insulin receptor gene, *IGF2* insulin growth factor 2 gene, *CI* conference interval, *WC* waist circumference, *BMI* body mass index, *SE* standard error.

All the analysis adjusted for bisulfite batch, province, gender, physical activity level, the frequencies of meat, vegetables, fruit, milk, smoking and alcohol use.

## Discussion

We found that DNA methylation in *INSR* might mediate the association between prenatal exposure to the Chinese great famine and waist circumference in adulthood. To our knowledge, epigenetic study of the Chinese great famine and waist circumference in later life is limited. Our findings suggest that maternal malnutrition during gestation may permanently change DNA methylation among offspring and link with the offspring’s waist circumference in adulthood. Which provided further evidence for hypotheses that epigenetic factors mediated the associations between exposure to an adverse environment during early development and adulthood health outcomes. Indicating that adequate nutrition supply to pregnant women before and during pregnancy seems a promising strategy to prevent central obesity in the next generation.

In the current study, we observed that individuals who were exposed to the Chinese great famine in fetal stage had a higher waist circumference in later life than those who did not experience the famine. The finding is similar to that of a study among 2,414 people born around the Dutch famine, in which, prenatal famine exposure increased waist circumference at age 50 in women but not in men^18^. Further analysis stratified by sex in the current study demonstrated that there was no statistically significant difference between men and women in the famine-waist circumference association. This indicates that the impact of short- and long-term famine exposure during fetal stage may differ in men. It is possible that the current study cannot detect gender difference due to smaller sample size and hence lower statistical power. However, the Chinese great famine lasted much longer than the Dutch famine, and the majority participants of the current study were recruited from Anhui province, which experienced the most severe famine during 1959–1961. The impact of famine should be more easily detected. Future large-scale studies are warranted to explore gender differences in the Chinese great famine’s impact on health conditions.

A significant association between prenatal exposure to the Chinese great famine and DNA methylation in *INSR* gene was found in the present study. Compared with the non-exposed group, those with prenatal exposure to the Chinese great famine had an average of 5.0% higher methylation level in *INSR*. This effect is larger than that reported in the Dutch famine study in similar DNA region, in which prenatal exposure to 6 months’ famine was associated with 3.8% higher methylation level in the *INSR* gene^12^. Again, this difference may be attributed to the much longer duration and more extreme severity of the Chinese great famine. In China, there are also regions with various severity of famine exposure during the 1959–1961. Future studies exploring a dose–response relationship between famine severity and DNA methylation level in the *INSR* and other genes are warranted.

The current study also identified that DNA methylation at the CpG7 site of the *INSR* gene was positively associated with waist circumference. In the Dutch famine study, prenatal malnutrition-associated DNA methylation regions in the *INSR* was correlated with birth weight, but not waist circumference^12^. Given the larger effect of the Chinese great famine on *INSR* gene methylation, it is not surprising to detect such association between famine-associated methylation and adult waist circumference in our study. Although further studies are needed to validate the finding in an independent Chinese sample, our finding may have a biological relevance. The CpG 7 unit lies in the promoter region of *INSR*, previous study had found that methylation of this unit may have reduced the expression of *INSR*^12^. The *INSR* encodes a member of the receptor tyrosine kinase family of proteins, which is processed to generate alpha and beta subunits of a heterotetrametric receptor. This receptor binds insulin or other ligands to activate the insulin signaling pathway, and subsequently regulates glucose uptake and release and is involved in lipids synthesis and storage. Loss-of-function mutations in this gene cause severe insulin resistance syndromes manifested by several studies^19,20^.

Finally, differential methylation at the CpG 7 unit of *INSR* associated with famine exposure contributed to a fairly large proportion (32.0%) of the association between prenatal famine exposure and adult waist circumference. Still, a large proportion of the famine-waist circumference association remains unexplained. Future genome-wide epigenetic studies are warranted to identify more methylation regions underlying such association.

Our study represents epigenetic research on the prenatal exposure to the Chinese great famine and central obesity in adulthood. The Chinese great famine lasted for 3 years and affected the entire main land China. It provides an opportunity to study the impact of long-term and severe malnutrition on epigenetic changes in human genome. In addition, a representative sample of residents from a famine-stricken region and a region with moderate famine exposure has been used. This enhanced the generalizability of our findings. Several limitations should be mentioned. Firstly, the percentage of mediation reported in the current study was estimated by the statistical model, which might overestimate the real effect of DNA methylation changes on WC. Secondly, DNA methylation data was measured from peripheral whole blood, which might be not the most relevant tissues to study for waist circumference. However, previous DNA methylation data from multiple tissues from the same donors showed a broad agreement in DNA methylation patterns of the CpG units in whole blood and various fat deposits^21,22^. Thirdly, the current study only selected a candidate locus, and did not conduct a genome-wide DNA methylation difference study, nor did perform a blood cell count, which limited the interpretation of the data. Forth, the statistically significant mediation observed in the current study did not clarify the mechanism, but it does provide possible pathways to explore in future. Fifth, age difference between prenatal famine exposure and non-exposed groups might be another important limitation in the current study. Due to the independent variable (famine-exposed/non-exposed groups) was defined by participants’ birthdates, which determines the age of participants. As a result, the ages of participants with prenatal famine exposure did not overlap the ages of participants without prenatal famine exposure. However, in the current study, we combined the pre-famine exposed group and post-famine exposed group as the non-exposed group. Thus, the mean age of exposed group and non-exposed group was similar. Sixth, the sample size is limited, and the level of statistical evidence is moderate. Thus, findings from the current study need to replicate in another Chinese famine cohort. Additionally, the information of smoking status was raw in the current study, which could not control totally the effect of smoking status on the association.

## Methods

### Study participants

The GRECF study was designed to investigate the impact of the Chinese great famine on the human genome and its subsequent impact on chronic conditions in later adulthood. The study has been introduced in details in a previous publication^15^. Briefly, the GRECF study recruited 790 participants from two neighboring provinces, Anhui and Jiangxi, in China through multi-stage clustered random sampling. Famine was most intense in Anhui province, while Jiangxi province was moderately affected^23, 24^. A random sample of 264 subjects were selected for DNA methylation assay. After excluding 29 subjects with extreme values of the mean DNA methylation level (> 3 standard deviation), 235 subjects were enrolled into the final analysis.

### Famine exposure

Food shortage gradually occurred in late 1958^25^. For the purpose of this study, we selected the start date of the Chinese great famine as January 1st, 1959 and the end date as December 31st, 1961^14^. Thus, subjects who were born between October 1st, 1959 and September 30th, 1961 were included as the prenatal famine exposed group, those who born between October 1st, 1962 and September 30th, 1964 were defined as post-famine exposed group, and those who born between January 1st, 1958 and December 31st, 1958 were categorized as the pre-famine exposed group. We combined the pre-famine exposed group and post-famine exposed group as the non-exposed group. Due to the Chinese great famine lasted from autumn of 1958 to autumn of 1962, especially during 1959–1961, subjects who born between October 1st, 1961 and September 30th, 1962 were excluded from the current study because they might also exposure to famine during second or third trimester of pregnancy^26^.

### Waist circumference, BMI and covariates

Waist circumference was measured to the nearest 0.1 cm by trained staff using a tape at the level of the umbilicus. Waist circumference was measured twice for each participant, and the average of the two measurements was used for the current analysis. BMI was calculated by dividing weight in kilograms by square of the height in meters (kg/m^2^).

Age was obtained from the National Resident Registration System. Other demographic and health behavioral information, including gender, smoking, and drinking, were collected based on self-report. Smoking status was categorized as ‘smoking less than 400 cigarettes’ or ‘smoking ≥ 400 cigarettes’ in the past year. Drinking status was classified as ‘current drinking’ and ‘not current drinking’. Physical activity was measured by the International Physical Activity Questionnaire Short Form (IPAQ-SF), and participants were categorized as having ‘vigorous’ or ‘non-vigorous’ physical activity^27^. Dietary intake of meat, vegetable, fruit, and milk was collected by the Chinese Food Frequency Questionnaire^28^. The intake frequency was divided into ‘< 1 time/day’ and ‘≥ 1 times/day’ for each food item.

### DNA methylation of the INSR and IGF2 genes

Genomic DNA was extracted from peripheral white blood cells using the salting-out method^29^. The EZ 96- methylation kit (Zymo Research) was used to bisulfite converted DNA. The conversion rate is greater than 99%. The Sequenom’s MassARRAY system (Sequenom, San Diego, CA) was used to quantify DNA methylation levels at CpG sites of the *INSR* and *IGF2* genes using the manufacturers’ protocol on a 384-well plate. Primers of the *INSR* and *IGF2* genes were designed using the Epidesigner online application (https://epidesigner.com/start3.html). Details of the primers and the CpG sites are presented in supplementary Tables S3 and S4.

DNA methylation of the *INSR* and *IGF2* genes in the current study was measured by the EpiTYPER, which is a mass spectrometry-based bisulfite sequencing method that enables region-specific DNA methylation analysis in a quantitative and high-throughput fashion^30^. We totally followed the protocol of EpiTYPER to perform the measure the methylation of *INSR* and *IGF2* genes. Sequenom’s EpiTYPER protocol includes the method and procedure of methylation data measurement^31^. To control the quality of measurement, we included a positive control (100% methylated) and a negative control (0%-methylated) for each run. All the samples were assayed in triplicate, if the triplicate methylation measurements had an extreme value ≥ 3 standard deviation, all data for the sample involved were discarded. The call rates over 85% for all CpG units. Among the 9 CpG sites at *INSR*, 7 CpG sites were measured individually, and two sites (CpG1 and CpG4) were combined and measured, because their fragment masses were so similar that they could not be distinguished. Among the 8 CpG sites at *IGF2*, 2 CpG sites (CpG6 and CpG7) were measured simultaneously because they could not be resolved due to their close proximity, and the others were measured individually. The overall methylation level is the arithmetic average of all CpG sites.

### Statistical analysis

The SPSS 20.0 and R software were used to perform statistical analyses. Continuous variables are presented as means ± standard deviations, and categorical variables are presented as percentages. Comparisons between famine exposed and non-exposed groups were conducted by Chi-square tests for categorical variables and two-independent samples *t*-tests for continuous variables. The normality test showed in Table S5.

Association between famine exposure and waist circumference was assessed by a multivariate linear regression model. A linear mix effect model was used to evaluate the association of DNA methylation with famine exposure, and the bisulfite patch was treated as a random effect in the model. Such analysis was performed for the overall methylation level and the methylation level at each CpG site. A Bonferroni corrected p value of 5.56 × 10^−3^ (correcting for 9 tests: 0.05/9 = 5.56 × 10^−3^) was used to determine significant CpG sites associated with prenatal famine exposure. Significant CpG site was further tested for association with waist circumference using the linear mix effect model. The “mediation” package of R software was used to analyze the mediation effect of DNA methylation at the significant CpG sites on the association between prenatal famine exposure and waist circumference in adulthood^32^. All the regression analyses controlled for gender, province, smoking, drinking, physical activity, and frequencies of meat, vegetable, fruit, and milk consumption. The detailed procedures for mediation model were listed below:

$$\begin{aligned} & {\text{model}}.{\text{m}} < - {\text{lm}}( {{\text{m}}\,\sim \,{\text{x}} + {\text{c}},{\text{data}} = {\text{dat}}} ) \\ & {\text{model}}.{\text{y}} < - {\text{lm}}( {{\text{y}}\,\sim \,{\text{x}} + {\text{m}} + {\text{c}},{\text{data}} = {\text{dat}}} ) \\ & {\text{out}}.{1} < - {\text{mediate}} ( {\text{model}}.{\text{m}},{\text{model}}.{\text{y}}, {\text{sims}} = {5}00,{\text{boot}} = {\text{ture}},{\text{treat}} = {\text{mediate}} = {``} {\text{m}} {{''}} ) \\ & {\text{summary}}\,( {{\text{out}}.{1}} ) \\ \end{aligned}$$

A power analysis was performed to assess the statistical power for examining the difference of DNA methylation between the prenatal famine exposed group and non-exposed group. The mean methylation level of *INSR* was 0.434 [standard deviation (SD) = 0.092) in the non-exposed group and 0.467 (SD = 0.070) in the prenatal exposed group. At a two-tailed alpha of 0.05, we had 85.5% power to detect the difference.

### Ethics statement

This study was approved by the Institute Review Board at the Peking University Health Science Center. Informed consent was obtained from all study participants. We confirmed that all methods were performed in accordance with relevant guidelines and regulations.

## Supplementary information

Supplementary information.

## Data Availability

The datasets generated during the current study are available from the corresponding author on reasonable request.
