# Supplementary material for: DNA methylation of the INSR gene as a mediator of the association between prenatal exposure to famine and adulthood waist circumference
Source: Sci Rep. 2020 Jul 22;10:12212. doi: 10.1038/s41598-020-69120-w (PMC7376170; doi:10.1038/s41598-020-69120-w)
Supplement: Supplementary file 1 — Supplementary information. [file 41598_2020_69120_MOESM1_ESM.docx]

**Higher DNA methylation of INSR gene mediates association between prenatal famine exposure and adulthood waist circumference**

Zhenghe Wang, Jieyun Song, Changwei Li, Yanhui Li, Luqi Shen, Bin Dong, Zhiyong Zou, Jun Ma

**Supplemental materials**

**Table S1**. The association between prenatal exposed to famine and adult WC stratified by sex

| Sex | Non-exposed group | Prenatal famine exposed group | *t* | *P* |
| --- | --- | --- | --- | --- |
| Male | 84.74±8.42 | 87.81±9.58 | 1.79 | 0.061 |
| Female | 81.20±8.66 | 83.91±8.67 | 1.66 | 0.072 |

Table S2. DNA methylation and prenatal exposure to famine stratified by region

| Genes locus | Exposed | Non-exposed | *d*^a^ | Effect size | *P*-value^b^ | *P-*value^c^ |
| --- | --- | --- | --- | --- | --- | --- |
|  | group | group |  |  |  |  |
| *INSR* |  |  |  |  |  | <0.001 |
| Anhui | 46.2±4.7 | 43.5±7.4 | 2.6 | 0.21 | 0.018 |  |
| Jiangxi | 47.3±9.1 | 43.3±10.9 | 4.0 | 0.20 | 0.062 |  |
| *IGF2* |  |  |  |  |  | <0.001 |
| Anhui | 43.4±7.3 | 38.7±7.3 | 4.7 |  | 0.025 |  |
| Jiangxi | 42.1±6.3 | 40.0±7.1 | 2.1 |  | 0.164 |  |

Abbreviations: *d,* Difference; *INSR*, insulin receptor gene; *IGF2*, Insulin growth factor 2 gene;

^a^ Average absolute difference in DNA methylation between exposed group and non-exposed group.

^b^ Independent-samples t-test to compare the difference between exposed group and non-exposed group.

^c^ Interaction analysis between region and famine exposure.

**Table S3**. The detailed information of EpiTYPER primers

| Target gene | Locus (NCBI36/hg18) | Primers | Strand | T(℃) | Sequences |
| --- | --- | --- | --- | --- | --- |
| *INSR* | chr19:7110130-7110574 | Forward (5'-3') | + | 58 | TGATTTTATTTTTAGGAGGTTTTTAGA |
|  |  | Reverse (5'-3') |  |  | ACTCACTACAACCTCTACCTCCCAA |
| *IGF2* | chr11: 2126035-2126372 | Forward (5'-3') | + | 58 | TGGATAGGAGATTGAGGAGAAA |
|  |  | Reverse (5'-3') |  |  | AAACCCCAACAAAAACCACT |

T, Annealing temperature; +, Forward; *INSR*, insulin receptor gene; *IGF2*, Insulin growth factor 2 gene;

**Table S4**. The detailed information about the genomic sites that methylation has been quantified

| Sites | Locus |
| --- | --- |
| *INSR* |  |
| CpG 1 | chr19:7110208 5'pad=0 3'pad=0 strand=- |
| CpG 2 | chr19:7110217 5'pad=0 3'pad=0 strand=- |
| CpG 3 | chr19:7110260 5'pad=0 3'pad=0 strand=- |
| CpG 4 | chr19:7110345 5'pad=0 3'pad=0 strand=- |
| CpG 5 | chr19:7110361 5'pad=0 3'pad=0 strand=- |
| CpG 6 | chr19:7110389 5'pad=0 3'pad=0 strand=- |
| CpG 7 | chr19:7110423 5'pad=0 3'pad=0 strand=- |
| CpG 8 | chr19:7110454 5'pad=0 3'pad=0 strand=- |
| CpG 9 | chr19:7110540 5'pad=0 3'pad=0 strand=- |
| *IGF2* |  |
| CpG 1 | chr11: 2126041 5'pad=0 3'pad=0 strand=- |
| CpG 2 | chr11: 2126057 5'pad=0 3'pad=0 strand=- |
| CpG 3 | chr11: 2126060 5'pad=0 3'pad=0 strand=- |
| CpG 4 | chr11: 2126154 5'pad=0 3'pad=0 strand=- |
| CpG 5 | chr11: 2126202 5'pad=0 3'pad=0 strand=- |
| CpG 6. | chr11: 2126251 5'pad=0 3'pad=0 strand=- |
| CpG 7 | chr11: 2126296 5'pad=0 3'pad=0 strand=- |
| CpG 8 | chr11: 2126312 5'pad=0 3'pad=0 strand=- |

-, Reverse

**Table S5**. Tests of Normality used the method of the Kolmogorov-Smirnov

| Variables | Exposed group |  | Non-exposed group |
| --- | --- | --- | --- |
| WC | 0.810 |  | 0.785 |
| BMI | 0.806 |  | 0.724 |
| *INSR* | 0.159 |  | 0.999 |
| CpG 1 | 0.104 |  | 0.329 |
| CpG 2 | 0.157 |  | 0.775 |
| CpG 3 | 0.213 |  | 0.633 |
| CpG 4 | 0.104 |  | 0.329 |
| CpG 5 | 0.080 |  | 0.371 |
| CpG 6 | 0.383 |  | 0.706 |
| CpG 7 | 0.176 |  | 0.247 |
| CpG 8 | 0.122 |  | 0.949 |
| CpG 9 | 0.135 |  | 0.711 |
| *IGF2* | 0.605 |  | 0.996 |
| CpG 1 | 0.271 |  | 0.254 |
| CpG 2 | 0.081 |  | 0.556 |
| CpG 3 | 0.062 |  | 0.581 |
| CpG 4 | 0.055 |  | 0.753 |
| CpG 5 | 0.083 |  | 0.065 |
| CpG 6&7 | 0.091 |  | 0.818 |
| CpG 8 | 0.785 |  | 0.358 |
